# Recent advances in understanding context-dependent mechanisms controlling neurotrophin signaling and function

**DOI:** 10.12688/f1000research.19174.1

**Published:** 2019-09-19

**Authors:** Mark Bothwell

**Affiliations:** 1 Department of Physiology & Biophysics, University of Washington Medical Center, Seattle, WA, 98195-7290, USA

**Keywords:** Brain derived neurotrophic factor, neurotrophin, BDNF, TrkB, p75NTR, synaptic plasticity, depressive disorders, memory, sortilin, SorCS2

## Abstract

Complex mechanisms control the signaling of neurotrophins through p75 ^NTR^ and Trk receptors, allowing cellular responses that are highly context dependent, particularly in the nervous system and particularly with regard to the neurotrophin brain-derived neurotrophic factor (BDNF). Recent reports describe a variety of sophisticated regulatory mechanisms that contribute to such functional flexibility. Mechanisms described include regulation of trafficking of alternative BDNF transcripts, regulation of post-translational processing and secretion of BDNF, engagement of co-receptors that influence localization and signaling of p75 ^NTR^ and Trk receptors, and control of trafficking of receptors in the endocytic pathway and during anterograde and retrograde axonal transport.

## Introduction

Neurotrophins are a small orthologous family of growth factors, consisting of nerve growth factor (NGF), brain-derived neurotrophic factor (BDNF), neurotrophin-3 (NT3), and neurotrophin-4 (NT4) in mammals. Neurotrophins have a remarkably wide range of critically important functions in both neural and non-neural tissues, and this diversity of function is achieved by signaling mechanisms that are highly context dependent. This review will explore recent advances in understanding how such context-dependent signaling is achieved, focusing on two of the most extensively studied functions of neurotrophins: regulation of neuronal survival and axon and dendritic growth in the developing peripheral nervous system and regulation of synaptic function in the context of learning and memory in the adult central nervous system (CNS). However, the principles and mechanisms discussed will be relevant for understanding other neurotrophin functions as diverse as mediation of the effects of anti-depressant drugs ^1, 2^, regulation of cardiac development ^3^, hypothalamic control of energy balance ^4^ and oxytocin-dependent maternal behavior ^5^, control of pancreatic glucose-induced insulin secretion ^6^, and control of insulin-dependent glucose uptake ^7^.

Two classes of receptors mediate neurotrophin effects. The 75 kDa neurotrophin receptor (p75 ^NTR^) binds all four neurotrophins, whereas orthologous members of the Trk gene family—TrkA, TrkB, and TrkC—are more selective. NGF and NT3 activate TrkA, BDNF and NT4 activate TrkB, and NT3 activates TrkC. Trk receptors are canonical receptor tyrosine kinases. p75 ^NTR^ is a member (and indeed was the first-discovered member) of the tumor necrosis factor (TNF) receptor superfamily and the subclass of those receptors known as death receptors because they contain a cytoplasmic death domain that mediates key signaling effects ^8^.

Further complexity in neurotrophin signaling results from the differential signaling capacity of mature neurotrophins and the longer precursor proteins (proneurotrophins) from which mature neurotrophins are released proteolytically. Proteolytic processing of proneurotrophins by prohormone convertases in the secretory pathway is not always complete, so proneurotrophins are sometimes secreted, and secreted neurotrophins may be variably released from proneurotrophins following secretion by membrane metalloproteinases. Both proneurotrophins and mature neurotrophins bind and activate p75 ^NTR^, whereas only mature neurotrophins can bind and activate Trk receptors ^9^. Consequently, the extent of proteolytic processing of proneurotrophins, either in the secretory pathway or following secretion, determines the balance of p75 ^NTR^ versus Trk signaling, a phenomenon that has received particular attention for BDNF/pro-BDNF signaling through p75 ^NTR^ and TrkB at CNS synapses ^10^. Remarkably, the cleaved pro-domain peptide of pro-BDNF has substantial stability and is stored in vesicles and co-secreted with BDNF ^11^. This feature and the technical issue that antibodies against BDNF do not discriminate between pro-BDNF and mature BDNF, while antibodies against the pro-domain do not discriminate between pro-BDNF and its cleaved pro-domain peptide, have contributed to uncertainty and controversy concerning the forms in which BDNF is secreted. Remarkably, the pro-domain peptide itself may engage in p75 ^NTR^-dependent signaling ^12– 14^, although the physiological relevance of this phenomenon remains to be fully explored.

This review will explore recent findings concerning four themes that figure prominently in neurotrophin studies. The first two themes are explored below as separate topics, whereas the remaining two themes cut across all topics. Theme 1: in cells that express both p75 ^NTR^ and Trk receptors, function of the two receptor systems is linked, but the two receptor systems often function oppositely. Theme 2: in the nervous system, neurotrophins may signal in an anterograde manner (from axon terminals to innervated targets), in a retrograde manner (from innervated target to innervating neuron), or in an autocrine manner and, in some systems, may use all three modes of action simultaneously. Theme 3: neurotrophin function is dependent on exquisitely controlled intracellular trafficking of neurotrophins and neurotrophin receptors. Theme 4: the nature of the signaling functions of p75 ^NTR^ and Trk receptors in neurons differs in different cellular contexts. The modes of signaling may differ in different types of neurons, at different stages of development of a neuron, and in somatic, dendritic, and axonal cell compartments of neurons.

## Opposing actions of p75 ^NTR^ and Trks

That p75 ^NTR^ and Trk receptors often function oppositely has been referred to as the yin and yang of neurotrophin action ^15^. Developing sympathetic neurons, for example, express both TrkA and p75 ^NTR^. For these neurons, activation of TrkA by NT3 promotes axon growth, and activation of TrkA by NGF promotes axon growth and neuronal survival ^16^. On the other hand, activation of p75 ^NTR^ by BDNF/pro-BDNF promotes axonal pruning and cell death ^17, 18^. In the adult CNS, BDNF-dependent activation of TrkB strengthens synaptic function and promotes long-term potentiation whereas BDNF-dependent activation of p75 ^NTR^ weakens synaptic function and promotes long-term depression ^15^. However, seemingly, nothing is ever simple in neurotrophin biology, as, under some circumstances, p75 ^NTR^ signaling can promote neuronal survival ^19^ and Trk signaling can promote neuronal cell death ^20, 21^ (discussed more fully below).

**Figure 1.**
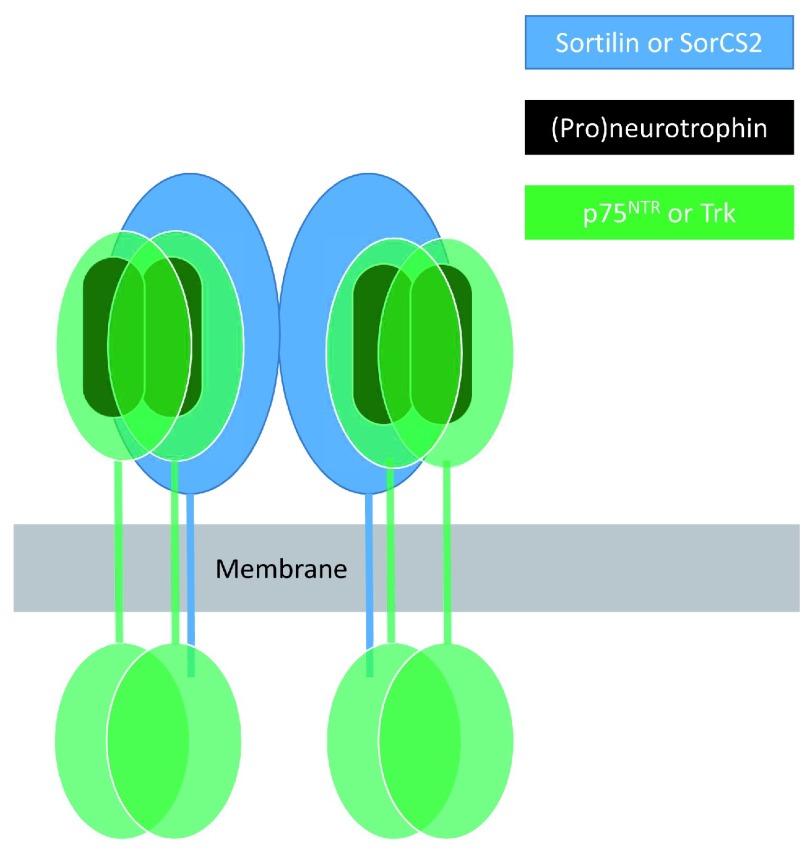
VPS10p-domain co-receptors of p75 ^NTR^ and Trk receptors. At least two members of the VPS10p-domain family of membrane proteins—sortilin and SorCS2—bind neurotrophins and their p75 ^NTR^ and Trk receptors. Neurotrophins bind as dimers to dimeric forms of p75 ^NTR^ and Trks. SorCS2 exists as a dimer, each subunit of which can apparently engage a neurotrophin (or proneurotrophin) dimer and associated dimeric p75 ^NTR^ or Trk receptor ^18^.

Functions of p75 ^NTR^ and TrkB are influenced by two co-receptors—sortilin and SorCS2—membrane proteins that are members of the VPS10p-domain protein family. The preferential interaction of proneurotrophins with p75 ^NTR^ is dependent on association of p75 ^NTR^ with sortilin or SorCS2 ^9, 22^, which make binding contacts with the neurotrophin pro-domains ^23^. Such interactions also mediate sortilin-dependent intracellular sorting of pro-BDNF into the regulated secretory pathway ^24^ and mediate p75 ^NTR^-dependent disassembly of hippocampal dendritic spines elicited by secreted cleaved BDNF pro-domain ^12^. In sensory neurons of the peripheral nervous system, association of sortilin with TrkA, TrkB, or TrkC promotes anterograde transport of these receptors to their axon terminal sites of action ^25^. In dendrites of hippocampal neurons, BDNF-dependent activation of TrkB required for dendritic spine formation and induction of synaptic long-term potentiation requires association of TrkB with SorCS2 whereas SorCS2 association with p75 ^NTR^ is required for pro-BDNF-dependent induction of synaptic long-term depression ^26^. Evidence suggests that sortilin or SorCS2 forms ternary complexes with neurotrophins or proneurotrophins and p75 ^NTR^ or Trk receptors ^22, 23, 27^. Figure 1 illustrates a speculative model for the nature of such ternary complexes.

## Anterograde, retrograde, and autocrine action

The first function ascribed to neurotrophins historically, regulation of neuronal survival by factors released by innervated targets, requires that neurotrophin receptors be localized to the axon terminus and, where the innervated targets are neurons, implies that neurotrophins are released from dendritic sites. On the other hand, for more recently characterized systems in which neurotrophins regulate synaptic function rather than neuronal survival, it is often initially unclear whether neurotrophins are released from dendrites to activate axonal receptors or whether neurotrophins are released from axons to activate dendritic receptors. Remarkably, not only do all of these scenarios come into play but a third scenario involving autocrine release and receptor activation in the same cell compartment plays an important role, as discussed below. Exquisitely complex mechanisms create ordered behavior from a potentially chaotic system by controlling neurotrophin release and receptor localization. I will summarize recent studies shedding light on these processes.

## Control of anterograde and retrograde receptor trafficking

In circumstances where neurotrophins secreted by the innervated target control neuronal survival, how does activation of receptors at the axon terminus influence required changes in nuclear gene expression when the somatic compartment containing the nucleus may be many centimeters distant from the axon terminus? An abundance of evidence indicates that a major mechanism mediating this effect is neurotrophin-dependent endocytic internalization of neurotrophin/Trk complexes at the axon terminus, followed by retrograde axonal transport of activated receptors in the vesicular complex ^28– 31^. This critically important transport of Trk receptors from axon terminus to soma must be balanced by a similar rate of transport of receptors translated in the soma to the axon terminus. For TrkA, one remarkable mechanism achieving this balance directly links arrival in the soma of retrogradely transported vesicles containing activated TrkA to anterograde transport of nascent TrkA. Nascent TrkA receptors are inserted into somatodendritic membranes and then endocytically internalized before anterograde axonal transport to the axon terminus, a process known as transcytosis. Endocytosis of nascent TrkA from somatodendritic membranes to initiate anterograde transport is triggered by tyrosine phosphorylation of TrkA by activated TrkA arriving through retrograde axonal transport ^32^. It is worth noting, however, that retrograde axonal transport is not the only mechanism that can deliver activated Trk receptors to the somatodendritic compartment. It is noteworthy that a transactivation mechanism causes activity of several G-protein-coupled receptors (GPCRs), including PACAP (pituitary adenylate cyclase-activating peptide) and adenosine receptors, to activate Trk receptors in the somatodendritic compartment ^33^. Therefore, it is interesting to speculate that GPCR signaling might enhance the accumulation of Trk receptors at axonal termini, rendering the capacity for retrograde signaling dependent on the cellular context.

Although retrograde axonal transport links activation of Trk receptors at the axon terminus to neuronal survival, activated Trk receptors acting locally contribute to the regulation of growth and arborization of the axon terminus, and mechanisms exist to segregate local and retrograde signaling modalities. For example, during sympathetic neuronal development, NT3 released along the path of axon growth sustains axonal elongation by activation of TrkA in a manner that does not require retrograde signaling to the soma, but NT3 is insufficient to sustain neuronal survival. Instead, activation of TrkA by NGF encountered when the axon reaches its final target sustains neuronal survival by retrograde signaling to the soma. How do NT3 and NGF engaging the same TrkA receptor signal locally in one case and at a distance in the other? One elegantly simple mechanism results from the stability of NGF/TrkA binding at the acidic pH of endosomes. Unlike NGF, NT3 dissociates from TrkA at acidic pH and cannot maintain the sustained activation of internalized TrkA required to convey signals to the cell soma ^34^. Additionally, a molecular switch mechanism causes NGF activation of TrkA to suppress subsequent activation of TrkA by NT3. This mechanism involves NGF/TrkA retrograde signaling, which transcriptionally upregulates Coronin-1. Coronin-1 suppresses the ability of NT3/TrkA complexes to release calcium ion from intracellular stores in the axonal compartment. In the absence of Coronin-1, sympathetic axons overshoot their targets ^35^.

In sympathetic neurons, association with TrkA with an axonally enriched non-translated transcript, Tp53inp2, is required for the NGF-dependent TrkA endocytosis event necessary to initiate retrograde axonal signaling supporting axon growth and cell survival ^36^. Whether similar mechanisms exist for TrkB and TrkC remains unknown. Sympathetic neurons express p75 ^NTR^ as well as TrkA, and, like TrkA, p75 ^NTR^ mediates both local functions at the axon terminus and functions in the cell soma requiring retrograde axonal signaling. Several mechanisms link functions of p75 ^NTR^ and TrkA. p75 ^NTR^ exhibits two distinct modes of signaling. Association of neurotrophins with p75 ^NTR^ dimers at the cell surface causes a conformation change of the receptors’ intracellular domains oppositely modulating signaling adapter proteins linking p75 ^NTR^ to regulation of the RhoA pathway and nuclear factor kappa B (NF-κB) and c-Jun N-terminal kinase (JNK) pathways ^37^. However, in sympathetic neurons, an alternative mode of p75 ^NTR^ signaling, leading to nuclear accumulation of NRIF (nuclear receptor-interacting factor) and cell death, involves γ-secretase-mediated cytoplasmic release of the intracellular domain (ICD) of p75 ^NTR^, which is promoted by neurotrophin-dependent activation of Trk receptors but is not directly promoted by neurotrophin binding p75 ^NTR^ ^38– 40^. p75 ^NTR^ signaling alternatively can activate cell survival-promoting pathways, such as the NF-κB pathway, or cell death-inducing pathways, such as the JNK pathway, and mechanisms determining these alternative responses are poorly understood. Modulation of γ-secretase-mediated release of the p75 ^NTR^ ICD fragment is one mechanism that may govern these responses, as pharmacologically blocking cytoplasmic release of the ICD fragment abolishes NF-κB activation and allows JNK-dependent induction of cell death in cerebellar granule cell neurons ^19^.

In addition to the Trk-dependent release of the p75 ^NTR^ ICD, several other mechanisms link Trk and p75 ^NTR^ signaling. Association of p75 ^NTR^ with TrkA increases the affinity of TrkA for NGF and blunts activation of TrkA by NT3 ^41, 42^. In sympathetic neurons, NGF-dependent activation of TrkA leading to Arf6 activation promotes trafficking of p75 ^NTR^ from intracellular vesicular stores to the cell surface. In contrast, NT3 activation of TrkA does not promote Arf6 activation and p75 ^NTR^ trafficking. Thus, NGF/TrkA signaling suppresses NT3/TrkA signaling, sharpening the developmental switch from NT3- to NGF-dependent functions ^43^. p75 ^NTR^ signaling at the axon terminus can locally regulate axonal growth cone collapse. However, p75 ^NTR^ also can signal retrogradely from the axon terminus to the soma to induce neuronal cell death. Although Trk activation promotes release of the p75 ^NTR^ ICD, loss of NGF-dependent TrkA activation in sympathetic neurons causes enhanced production of the ICD fragment and HDAC1-dependent retrograde trafficking of this fragment mediates retrograde axonal conveyance of the p75 ^NTR^ cell death signal ^44^. Similarly, enhanced release of the p75 ^NTR^ ICD fragment has been implicated as a mechanism responsible for neuronal cell death promoted by unliganded TrkA and TrkC in the developing peripheral nervous system ^20^.

## Synaptic functions of p75 ^NTR^ and Trks

Although all the neurotrophins and neurotrophin receptors function in the CNS, the most broadly important neurotrophin in the brain is BDNF, which influences many types of neural plasticity in diverse neuronal populations, including cortical and hippocampal neurons, through TrkB and p75 ^NTR^ receptors. BDNF function in the hippocampus, and particularly at the synapses of CA3 Schaffer collaterals on CA1 dendrites, has been extensively studied because BDNF interactions with TrkB and p75TR at hippocampal synapses are critical for long-term potentiation ^45, 46^, an experimental synaptic model for learning and memory. Unraveling these mechanisms is far from straightforward, as BDNF can be released by both pre-synaptic axon termini and post-synaptic dendrites, and the receptors are present both pre-synaptically and post-synaptically ^47^. Furthermore, peri-synaptic microglia, astrocytes, and oligodendroglia are all physiologically relevant sources of BDNF release at CNS synapses ^48– 50^. Although both pre-synaptic and post-synaptic action of BDNF has been described, a particularly thorough report from 2016 describes an autocrine mode of BDNF/TrkB signaling in CA1 dendritic spines involving NMDA receptor-dependent activation of CaMKII-dependent BDNF release ^51^.

Studies in Aplysia, an important model organism for the study of mechanisms of learning and memory, have revealed an important role for pre-synaptic autocrine neurotrophin action. Aplysia has a single neurotrophin gene ( *ApNT*) and a single Trk receptor gene ( *ApTrk*) ^52^. ApNT promotes 5HT-mediated consolidation of learning-related facilitation of a sensory neuron/motor neuron synapse. 5HT-dependent production of cAMP pre-synaptically promotes pre-synaptic release of ApNT, which activates pre-synaptic ApTrk driving phospholipase C-dependent calcium ion release, resulting in further elevation of cAMP by a calcium-dependent adenylate cyclase. This constitutes a positive feedback loop driving ApNT release and neurotransmitter (glutamate) release ^53^. Pre-synaptically released ApNT acting on post-synaptic ApTrk promotes post-synaptic ApNT release, which acts pre-synaptically. Thus, two neurotrophin feed-forward systems provide signal amplification for 5HT’s effects on synaptic function ^54^.

An important feature of BDNF function in mammalian dendrites is that dendritic BDNF is a product of peri-synaptic protein synthesis. BDNF transcription employs nine different promoters which, in concert with alternative splicing of 5′ untranslated regions (5′ UTRs), generate 22 different transcripts all encoding identical proteins ^55^. *In vitro* studies reveal that these 5′ UTRs differentially direct different transcripts to different subcellular domains ^56^. The differential regulation of the different promoters by neural activity, coupled with the different subcellular targeting of the different transcripts, allows complex regulation of neuronal responses. For example, transgenic mouse models reveal that different splice variants differentially control size of spines on apical versus basal CA1 dendrites ^57^. In concert with BDNF/TrkB action promoting the formation of hippocampal dendritic spines, pro-BDNF/p75 ^NTR^ action suppresses the formation of dendritic spines ^58^. As noted above, SorCS2 acts as a necessary co-receptor for both p75 ^NTR^ and TrkB acting oppositely to control the formation of hippocampal dendritic spines. Whether the differential engagement of p75 ^NTR^ versus TrkB by SorCS2 is an important point of regulation is a question that deserves investigation.

## Conclusions

Despite the impressive recent progress in revealing the extraordinary complexity of neurotrophin signaling functions, many unanswered questions remain. The manner by which neurotrophin-deprived Trk receptors promote the release of the proapoptotic p75 ^NTR^ ICD fragment remains mysterious, and the relative importance of signaling by intact cell-surface p75 ^NTR^ versus signaling by the cleaved p75 ^NTR^ ICD fragment in various contexts remains unclear. Indeed, many aspects of p75/Trk functional interactions remain unclear despite decades of study. In many cases, mechanisms governing neurotrophin receptor anterograde and retrograde trafficking have been characterized for a single Trk paralog and in a single neuronal cell type at a particular stage of development. It should be a priority to determine the extent to which such mechanisms apply to other Trk paralogs and in other neuronal cell types or other stages of development.
